# Prevention of Typhoid

**Published:** 1915-09

**Authors:** 


### Prevention of Typhoid.

Do not forget that, owing to the relatively greater progress of cities in sanitation, as compared with villages, isolated dwellings and especially hastily and cheaply constructed hotels and boarding houses occupied only during the summer months, typhoid is not mainly a vacational disease. It is even more plainly an early autumnal disease than in the old days when we all drank diluted sewage, and even more plainly not explainable on climatic grounds, except in the sense that summer tempts us to places where the water is worse than the average.

Vaccination has rendered typhoid almost as preventable as small-pox, though the protective value of the vaccination is less permanent and not so nearly absolute. Do not be too much afraid of seeming mercenary. Advise your patients and acquaintances—not forgetting your family and yourself—to be vaccinated against typhoid, unless they have already had the disease or have been vaccinated within two years. If necessary, vaccine can be obtained free from the Health authorities in most places but we see no reason in encouraging either public or private charity for well-to-do persons, in this condition more than in the event of an actual infection. Remember, however, that, as long ago threshed out for vaccinia, it is neither advisable nor safe to vaccinate if an infection has already occurred.

