# Correction

**Published:** 1915-10

**Authors:** 


Please accept our apology for several mistakes in printing in the September issue. With one exception, these would lead to no confusion. In the editorial on the Prevention of Typhoid, however, we were made to say that it was **not** mainly a vacational disease—the exact reverse of our opinion.

